# Cadmium-Tolerant Rhizospheric Bacteria of the C_3_/CAM Intermediate Semi-Halophytic Common Ice Plant (*Mesembryanthemum crystallinum* L.) Grown in Contaminated Soils

**DOI:** 10.3389/fpls.2022.820097

**Published:** 2022-03-08

**Authors:** Paulina Supel, Marta Śliwa-Cebula, Zbigniew Miszalski, Paweł Kaszycki

**Affiliations:** ^1^Department of Plant Biology and Biotechnology, Faculty of Biotechnology and Horticulture, University of Agriculture in Kraków, Kraków, Poland; ^2^Malopolska Centre of Biotechnology, Jagiellonian University, Kraków, Poland; ^3^W. Szafer Institute of Botany, Polish Academy of Sciences, Kraków, Poland

**Keywords:** heavy metal stress, Crassulacean acid metabolism, bioremediation, soil microbiota, common ice plant, salt tolerance, halophytic bacteria

## Abstract

The common ice plant, *Mesembryanthemum crystallinum* L., has recently been found as a good candidate for phytoremediation of heavy-metal polluted soils. This semi-halophyte is a C_3_/CAM (Crassulacean acid metabolism) intermediate plant capable of tolerating extreme levels of cadmium in the soil. The aim of the work was to obtain and characterize novel, Cd-tolerant microbial strains that populate the root zone of *M. crystallinum* performing different types of photosynthetic metabolism and growing in Cd-contaminated substrates. The plants exhibiting either C_3_ or CAM photosynthesis were treated for 8 days with different CdCl_2_ doses to obtain final Cd concentrations ranging from 0.82 to 818 mg⋅kg^–1^ of soil d.w. The CAM phase was induced by highly saline conditions. After treatment, eighteen bacterial and three yeast strains were isolated from the rhizosphere and, after preliminary Cd-resistance *in vitro* test, five bacterial strains were selected and identified with a molecular proteomics technique. Two strains of the species *Providencia rettgeri* (W6 and W7) were obtained from the C_3_ phase and three (one *Paenibacillus glucanolyticus* S7 and two *Rhodococcus erythropolis* strains: S4 and S10) from the CAM performing plants. The isolates were further tested for Cd-resistance (treatment with either 1 mM or 10 mM CdCl_2_) and salinity tolerance (0.5 M NaCl) in model liquid cultures (incubation for 14 days). *Providencia rettgeri* W7 culture remained fully viable at 1 mM Cd, whereas *Rh. erythropolis* S4 and S10 together with *P. glucanolyticus* S7 were found to be resistant to 10 mM Cd in the presence of 0.5 M NaCl. It is suggested that the high tolerance of the common ice plant toward cadmium may result from the synergic action of the plant together with the Cd/salt-resistant strains occurring within rhizospheral microbiota. Moreover, the isolated bacteria appear as promising robust microorganisms for biotechnological applications in bio- and phytoremediation projects.

## Introduction

The problem of soil degradation and deterioration is a consequence of anthropogenic activities such as manufacturing, fast industrial development and incorrect spatial planning, excessive and imbalanced natural resources exploitation and smelting, and also improper agricultural practices such as poor water and soil management, application of fertilizers, pesticides and fungicides (Asrari, 2014; Karlen and Rice, 2015). Almost 24% of world land area is considered as degraded (Karlen and Rice, 2015). The most common factors responsible for soil degradation are organic and inorganic chemicals. The first group consists of aliphatic and aromatic hydrocarbons and their derivatives, whereas the second one refers to acids, bases, salts, biogenic elements causing eutrophication, heavy metals, as well as many other complexes and derivatives (Ashraf et al., 2010; Karlen and Rice, 2015; Khan et al., 2015; and the references therein).

Cadmium is considered to be one of the most hazardous substances occurring in soils at elevated concentrations, which originates predominately from fertilizer use (EU Directive, 2013; Nosek et al., 2019). It has no physiological nor biochemical function, and can reveal severe toxic action against microbes and higher organisms even at minor concentrations (Ali et al., 2013; Rizwan et al., 2017; El Rasafi et al., 2020). Its toxicity in plants results from alteration of essential nutrients uptake and transport, inhibition of cellular enzymes, production of reactive oxygen species (ROS) and water balance disruption (Nosek et al., 2019, 2020; El Rasafi et al., 2020). These changes in higher plants may lead to genotoxicity, malfunctions in respiration and photosynthesis, growth inhibition, chlorosis and root tip browning (Nagajyoti et al., 2010). Humans are the last link in a food chain and for that reason their health is particularly susceptible to the negative action of this heavy metal. The toxic effect of Cd is mostly manifested as damages in pulmonary, nervous and renal systems functioning (Aoshima et al., 2003).

Elevated soil salinity is yet another serious worldwide problem caused by excessive human activities such as deforestation, improper irrigation and fertilization (Ashraf et al., 2010; Liang et al., 2017). Approximately 20% of irrigated areas are affected with salinization caused by irrigation not only with seawater but also with water considered as good quality, which may still contain up to 500 mg⋅dm^–3^ of soluble salts (Zhang et al., 2018; Kataoka et al., 2021). Salinity is regarded as a common primary cause of stress in plants leading to increased osmotic pressure and water deficit, nutritional imbalance and accumulation of toxic ions in plant organs, mostly roots, and has strong negative influence on plant growth and development (Zanetti et al., 2019; Nosek et al., 2020).

In order to find the best possible solution to a problem of heavy metal contamination in saline soils, phytoremediation has been proposed as an efficient, inexpensive and environmentally friendly approach (Jesus et al., 2015; Liang et al., 2017). This method is constantly gaining more attention nowadays and numerous research-and-development projects tend to enable practitioners to cope with severe cases of polluted and degraded areas. Phytoremediation employs plants for extraction or detoxication and stabilization of pollutants that cannot be removed from soil with microbial biodegradation methods, such as heavy metals and salts (Jordan et al., 2002; Nosek et al., 2019). The main requirement for plants used for phytoextraction is an efficient uptake of a pollutant by roots and then translocation to shoots, which facilitates final removal of contamination with harvested biomass. This process should be favorably fast and characterized by the highest bioaccumulation yield (Ghnaya et al., 2005).

The common ice plant (*Mesembryanthemum crystallinum* L.) can serve as an interesting example of plants proposed for successful use in phytoremediation (Śliwa-Cebula et al., 2020). It is a semi-halophyte revealing high tolerance to many stresses including excess light, elevated salt concentrations, nutrients or water deficiency, low temperature, poor soil structure, and heavy metal presence (Adams et al., 1998; Kuznetsov et al., 2000; Libik-Konieczny et al., 2019). *M. crystallinum* is an edible member of *Aizoaceae* family originating from African Namib desert habitats (Winter et al., 1978; Kim et al., 2021), currently also cultivated as a food crop in China (Zhang et al., 2018). It is known as C_3_-CAM photosynthetic intermediate plant with a possibility to switch between the two ways of CO_2_ metabolism as an effect of exposure to stress factors (Winter et al., 2015; Libik-Konieczny et al., 2019). It reveals fast growth and strong root system development even at salt concentrations in soil reaching 1 M (Bohnert and Cushman, 2000; Kuznetsov et al., 2000; Śliwa-Cebula et al., 2020). NaCl is transported through roots to the specialized structures called epidermal bladder cells, which are developed on leaf and stem surfaces and are responsible for saline sequestration and osmotic pressure adjustment (Bohnert and Cushman, 2000; Kim et al., 2021). No significant toxic effect is visible up to 40% of salt content in plant dry mass (Amari et al., 2014). That unique feature makes the common ice plant a perfect model in saline phytoremediation (Kataoka et al., 2021). In turn, Ghnaya et al. (2005) first suggested that it may also be a perfect candidate in cadmium decontamination due to accumulation capabilities of considerable amounts of this element in tissues. Recent studies showed, however, that although *M. crystallinum* is resistant to very high cadmium concentrations, it employs mostly an exclusion strategy preventing excessive accumulation of this heavy metal in roots and shoots (Nosek et al., 2019, 2020; Śliwa-Cebula et al., 2020). This strategy enables the plant to protect its organs against toxicity, whereas the main portion of cadmium remains in the bulk soil. It has been experimentally demonstrated that this plant tolerated CdCl_2_ up to concentrations of 10 mM without any visible morphological toxicity symptoms and tended to accumulate Cd in tissues only at higher concentrations of this element (Nosek et al., 2019, 2020).

Root zone microbiota may play an important role in the resistance of plants to heavy metals (Zhang et al., 2018). Both endophytic and rhizospheric plant growth-promoting (PGP) microorganisms can considerably stimulate plant growth by affecting acquisition of nutrients, accumulation and/or biotransformation of pollutants thus enhancing plant tolerance to physiological stressors. They can also produce PGP hormones such as indole-3-acetic acid (IAA). Tissues or rhizosphere of *M. crystallinum* were shown to be colonized with microorganisms resistant to high saline concentrations and exhibiting PGP-traits, capable of N-fixation or P-solubilization (Zhang et al., 2018; Yaprak et al., 2019; Kataoka et al., 2021). Some of these strains were identified as belonging to genera *Halomonas, Bacillus, Planococcus*, *Microbacterium*, and *Streptomyces* (Zhang et al., 2018; Kataoka et al., 2021). The plant-beneficial effect of these microorganisms was manifested by significant root elongation and shoot mass increase (Yaprak et al., 2019), promotion of initial growth of seedlings (Kataoka et al., 2021) and improvement of salinity resistance of non-halophytic plant (Zhang et al., 2018).

The aim of this work was to obtain novel microbial strains that populate the rhizosphere of Cd-treated *Mesembryanthemum crystallinum*, a semi-halophyte and a cadmium excluder. Such Cd-resistant microorganisms were isolated from the root zones of the common ice plant performing either the C_3_ or CAM photosynthetic metabolism. The strains revealing considerable bioremediation potential toward cadmium contamination in soils and having capabilities to promote plant growth might contribute to the high cadmium tolerance of *M. crystallinum*.

## Materials and Methods

### Plant Material and Growth Conditions

*Mesembryanthemum crystallinum* L. seedlings were obtained from seeds upon cultivation in the greenhouse at a 16/8 day/night period, 300–350 μmol photons m^–2^ s^–1^ of photosynthetically active radiation (PAR), 25°C, and 60%/65% relative humidity. For cultivation, the commonly available universal soil substrate “Hawita Fruhstorfer soil type LD80” (Hawita Gruppe, GmbH, Germany) was used. The growth substrate characteristics were: pH value (CaCl_2_) 5.9; salt content, 1.0 g⋅dm^–3^; composition: white peat and clay, containing CaCO_3_ and a long-term delivery fertilizer to provide N, P, K and Mg at concentrations of 150, 150, 250, and 130 mg dm^–3^, respectively. Two-week old seedlings with a fully developed second leaf pair were planted in 1.2 dm^3^ pots filled with the soil substrate as described above, one plant per pot, and watered every day.

### Model Soil Cadmium Contamination Experiment

Six-week old plants were divided into two groups: (1) C_3_, irrigated with tap water and (2) CAM, irrigated with 0.4 M NaCl. After 2 weeks, the presence of C_3_ or CAM photosynthetic metabolism was confirmed by the measurements of the difference between malate concentration (Δ malate) at the beginning and at the end of the light phase (Guan et al., 2020). In this study the diurnal Δ malate concentration ranged from 2.7 to 4.6 mM and 18.2–23.8 mM for C_3_ and CAM-performing plants, respectively. Next, the 8-week old plants were treated with CdCl_2_ (Sigma-Aldrich, United States) solutions applied at the following concentrations: 0 (control), 0.01, 0.1, 1.0, and 10 mM administered daily as 10 cm^3^ doses per pot for consecutive 8 days. This experimental approach was analogous to the model elaborated by us earlier and previously described (Nosek et al., 2019, 2020; Śliwa-Cebula et al., 2020). Such treatment led to the final Cd doses of 0 (control), 0.8, 8.0, 80, and 800 μmol per pot, respectively, which were calculated as 0.82, 8.2, 82, and 818 mg Cd per kg of soil d.w. During the Cd-treatment microbial community frequency and diversity was monitored. At the last day of the experiment microorganisms from the root zone were isolated and cultivated to obtain pure cultures. After the experiment, the plants were collected and fresh/dry mass of shoots and roots was assessed with standard methods (Nosek et al., 2020; Kataoka et al., 2021).

Microbiological plates were thoroughly examined, then separate colonies were selected and subcultured. Pure strains were obtained with the standard streaking method (Zhang et al., 2018). For microbial cell population density determination, a modified Koch surface-plating method of aqueous suspensions of the soil substrate was used (Supel et al., 2018). Soil dry weight (d.w.) content was established with the standard drying method, and microbial frequency was calculated finally as colony forming units (CFU) per 1 g of soil d.w.

### Preliminary Microbial Tests

In the first experimental step, the strain isolates were preliminary tested for Cd resistance and saline tolerance in microtubes. For bacteria cultivation, liquid cultures with SNB (Standard Nutrient Broth) medium were used containing biomass density of OD_600_ > 1 (Optical Density, λ = 600 nm). For yeast a YCU (Yeast Common Use) medium was prepared with 1% glucose and the biomass was evaluated as OD_540_ > 1 (Zhang et al., 2018). Detailed composition of all the media used in the study is listed in Table 1. The following final concentrations of the tested compounds were applied: control (without CdCl_2_ and NaCl); 1 mM CdCl_2_; 10 mM CdCl_2_; 0.5 M NaCl; 0.5 M NaCl and 1 mM CdCl_2_; 0.5 M NaCl and 10 mM CdCl_2_. The experiment was carried out at the total medium volume of 0.25 cm^3^, 5 M saline and 1 M cadmium chloride solutions were administered at calculated quantities directly to microbial cultures and then the microtubes were cultivated for 14 days in a rotary-shaker incubator. After 0, 1, 4, 7, and 14 days, a 0.025 cm^3^ samples were collected and plated onto bacto agar-containing solid media. The test result was assessed as “positive” if the microbial growth was observed after incubation.

**TABLE 1:** Composition of liquid media used throughout the experiments (concentrations are given in grams per dm^3^).

Standard Nutrient Broth (SNB) medium (optimal)	Bushnell Haas Broth (BH) (minimal)	Yeast Common Use (YCU) medium (optimal)

Cultivation of bacteria	Cultivation of bacteria	Cultivation of yeast
	Magnesium sulfate—0.2	Calcium chloride—0.02
	Monopotassium phosphate–1.0	Dipotassium phosphate—1.0
Casein peptone—15.0	Ammonium nitrate—1.0	Ferric chloride—0.05
Yeast extract—3.0	Casein peptone—15.0	Yeast extract—3.0
NaCl—6.0	Amonium sulfate—60.0	Magnesium sulfate—6.0
	Monopotassium phosphate—10.0	Calcium chloride—2.4

### Cd Resistance and NaCl Tolerance Assessment in Model *in vitro* Microbial Tests

In the second step, the strains selected as “positive” in preliminary testing were cultivated in liquid culture in the SNB medium. After 4 days of cultivation on rotary shaker, the cultures were centrifuged at 5,500 rpm and supernatants suspended in Bushnell Haas medium (Fluka Analytical, United States) (Table 1) with the addition of 1% glucose to obtain the final biomass of OD_600_ = 0.5. Then, the tested compounds, i.e., NaCl and CdCl_2_, were added to the cultures at particular final concentrations. Six combinations were tested: control (without CdCl_2_ and NaCl); 1 mM CdCl_2_; 10 mM CdCl_2_; 0.5 M NaCl; 0.5 M NaCl, and 1 mM CdCl_2_; 0.5 M NaCl and 10 mM CdCl_2_. The experiment was carried out in Erlenmayer flasks containing total culture volumes of 20 cm^3^. Bacterial cell frequency was analyzed after 0, 1, 3, 5, 8, and 14 days of incubation.

### Microbial Strain Identification

The strain isolates were subjected to molecular proteomics identification with the Bruker Biotyper^®^ analyzer, an automated next generation microbial identification system (Bruker Daltonics GmbH & Co., KG, Germany) performing the Matrix-Assisted Laser Desorption/Ionization Time-Of-Flight Mass Spectrometry (MALDI TOF MS). Proteomic data represented by unique MS-based “fingerprint” peak profiles were matched with a Bruker data base MBT IVD Library version K (release 2020). Microbial identification accuracy was evaluated with an identification factor, IF, calculated as a logarithmic score between 0 and 3 to quantify the similarity to appropriate reference spectra within database entries. Each strain identity was scored with the resultant IF value and quantified as follows: high-confidence identification, for IF ≥ 2.0; low-confidence identification, for IF ranging from 1.7 to 1.99, and no organism identification possible, for IF < 1.7. The matched microbial species were then referred to the NCBI (The National Center for Biotechnology Information) taxonomy database maintained by NCBI/GenBank^1^ and each strain has been assigned a specific NCBI taxonomy identification number (NCBI:txid no.).

### Statistical Analysis

The Statistica 13.0 Pl (StatSoft Polska, Poland) software was used for statistical data evaluation. One-way analysis of variance (ANOVA) was applied and the significant differences between the results were analyzed with the Tukey’s test (*p* < 0.05).

## Results

### Model Soil Cadmium Contamination Experiment—Effect of Cd^2+^ and NaCl on Plants

**FIGURE 1:**
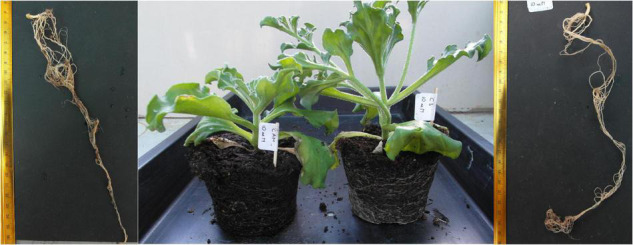
Morphology of *M. crystallinum* roots and plants grown in cadmium-contaminated soil (final Cd concentration of 818 mg kg^–1^ soil d.w.) irrigated either with 0.4 M NaCl (CAM phase, left) or with water (C_3_ phase, right). Ruler scale in (cm).

**FIGURE 2:**
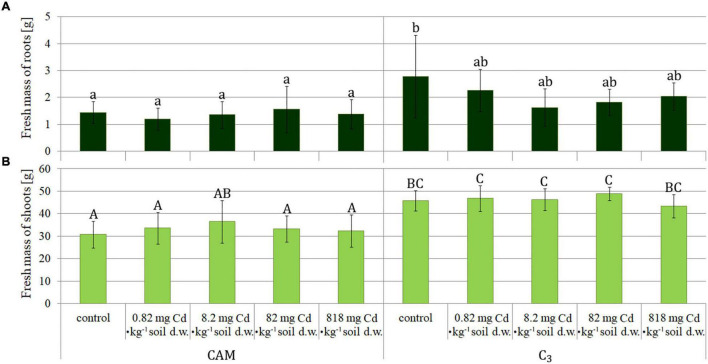
Fresh mass of *M. crystallinum* roots (A) and shoots (B) as assessed upon plant growth under variant Cd-treatment conditions. The concentrations given under the bars present the final Cd levels calculated based on 8-day administration with different doses of cadmium. Bars marked with the same letters indicate no statistical difference (*p* < 0.05); the results were statistically analyzed separately for shoots and roots.

**FIGURE 3:**
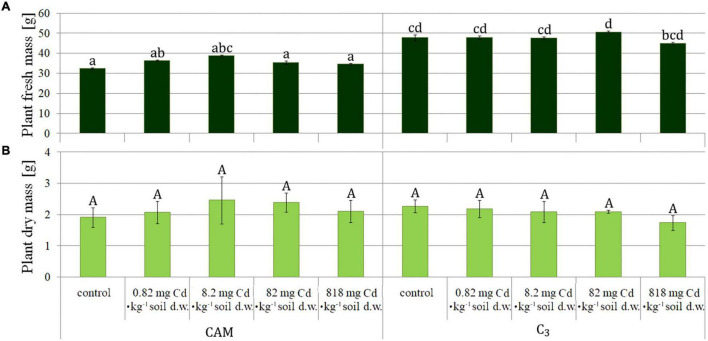
Comparison of fresh (A) and dry (B) mass of *M. crystallinum* plants performing either CAM or C_3_ photosynthetic metabolism upon growth under variant Cd-treatment conditions. The concentrations given under the bars present the final Cd levels calculated based on 8-day administration with different doses of cadmium. Bars marked with the same letters indicate no statistical difference (*p* < 0.05).

The plants were collected after 8 days of the experiment and then measured (Figure 1). The obtained results were similar for root fresh mass assessment in all the variants (Figure 2A), whereas statistical differences were observed for fresh mass of above-ground plant parts (shoots) (Figure 2B), indicating more expansive growth and a higher biomass formed under the C_3_ phase. Also, a comparison of total (either fresh or dry) plant mass (Figure 3) revealed that despite significant differences between fresh mass plants in C_3_ and CAM mode (Figure 3A), the dry mass was similar for all the cases (Figure 3B). These observations, supported with the general plant condition evaluation (Figure 1) suggest that plants in the CAM mode showed more compact growth, while the C_3_ plants had higher water content in the above-ground parts, thus developing more extensive forms. Also, the roots of C_3_ plants were more spread throughout the total volume of the substrate, whereas for the case of the CAM plants, they tended to grow tighter. Note that for each mode of plant photosynthetic metabolism, no differences between the applied Cd^2+^ concentrations were observed compared to the respective control variants. The detailed information on cadmium accumulation profiles of *M. crystallinum*, namely the Cd content in shoots and roots, can be found in a parallel study of Nosek et al. (2020).

### Model Soil Cadmium Contamination Experiment—Analysis of the Soil Microbiota and Strain Isolation

**FIGURE 4:**
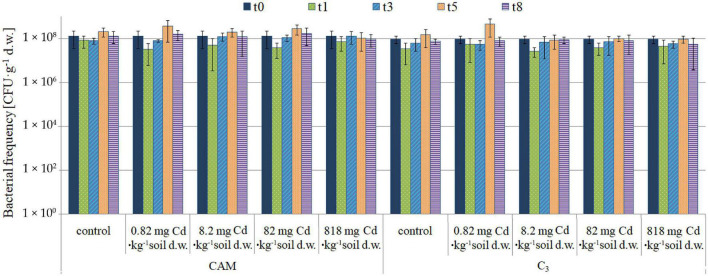
Population dynamics of the soil microbiota as observed upon the model experiment of *M. crystallinum* grown at variant Cd concentrations; t0, start of the experiment, t1–t8, days of cultivation. The concentrations given under the bars present the final Cd levels calculated based on 8-day administration with different doses of cadmium. No statistical differences (*p* < 0.05) were observed.

**FIGURE 5:**
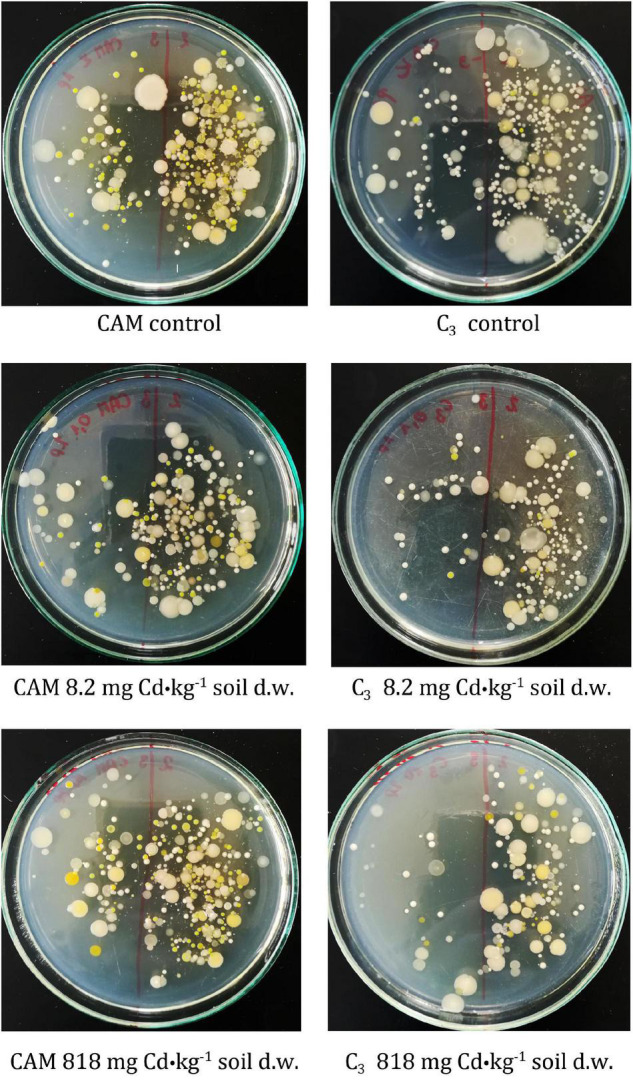
Comparison of *M. crystallinum* rhizospheric bacteria diversity based on colony morphologies after plating of microbiota-containing soil extracts. The tested *M. crystallinum* plants performed either C_3_ or CAM photosynthetic metabolism as induced by irrigation with tap water or saline solution (0.4 M NaCl), respectively. The samples were obtained from the root zones of control plants (top panel) and plants treated with Cd at final concentrations of 8.2 mg⋅kg^–1^ of soil d.w. (middle panel) or 818 mg⋅kg^–1^ of soil d.w. (bottom panel). The Cd concentrations were calculated based on 8-day administration with different doses of cadmium.

The first-stage experiment revealed high microbial rhizosphere colonization of about 10^8^CFU⋅g^–1^ d. w. of soil. Some decrease in bacterial frequency was observed after the first day of experiment for all variants, but the differences were not statistically significant. The microbiota population density remained constant during the 8-day experiment, and no significant changes in cell frequency were observed (Figure 4). Also, microbial community diversities as evaluated based on analysis of colony different morphotypes remained similar regardless of the applied cadmium or saline solutions (Figure 5).

Eighteen bacterial and three yeast strains were isolated from the plant rhizosphere, including one yeast strain isolated from the 0.1 mM CAM variant, nine bacterial and two yeast strains isolated from the 10 mM CAM variant and 9 bacterial strains from the 10 mM C_3_ variant. From the total of 21 isolates only 9 survived multiple microbiological passages, namely the strains denoted as S1 (yeast), and bacterial S4, S7, S10, S12, W1, W2, W6, and W7.

### Strain Identification Upon Preliminary Cd- and Salinity Tolerance Test

**FIGURE 6:**
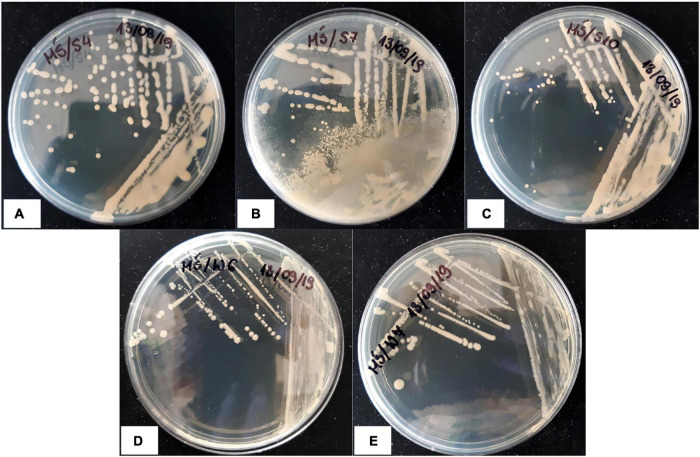
Colony morphotypes of Cd- and salt-tolerant bacterial isolates isolated from the root zones of Cd-treated *M. crystallinum*; **(A)** *Rhodococcus erythropolis* S4; **(B)** *Paenibacillus glucanolyticus* S7; **(C)** *Rhodococcus erythropolis* S10; **(D)** *Providencia rettgeri* W6; **(E)** *Providencia rettgeri* W7.

Nine strains obtained from the root zones of plants grown in the presence of CdCl_2_ were subjected to preliminary Cd and saline toxicity tests. The samples were incubated for 14 days, and microbial growth was analyzed at the start of the experiment and then after 1, 4, 7, and 14 days. The results of bacterial growth evaluation after 14-day treatment are shown in Table 2. Five bacterial strains were assessed as “positive” (S4, S7, S10, W6, and W7; see Figure 6 for colony morphotypes and Table 3 for detailed description) and became identified with the Bruker MALDI-TOF proteomic-based diagnostics as the following species (Table 3): *Rhodococcus erythropolis* (strains S4 and S10), *Paenibacillus glucanolyticus* (S7), and *Providencia rettgeri* (W6 and W7). These strains were selected for further experimenting and subjected to the full-scale tests.

**TABLE 2:** Results of preliminary Cd and saline toxicity test of the strains isolated from *Mesembryanthemum crystallinum* rhizosphere, obtained after 14 days of incubation.

Tested strain	Cultivation conditions					
	
	control	1 mM CdCl_2_	10 mM CdCl_2_	0.5 M NaCl - CdCl_2_	0.5 M NaCl 1 mM CdCl_2_	0.5 M NaCl 10 mM CdCl_2_
S1	+	−	−	+	−	+
S4	+	+	−	+	−	−
S7	+	+	−	+	+	−
S10	+	+	−	+	±	−
S12	−	−	−	−	−	−
W1	+	+	−	+	+	−
W2	+	+	+	+	+	−
W6	+	+	+	+	±	−
W7	+	+	−	+	±	−

*“+” strain growth observed in all the repetitions, “±” strain growth observed in at least one repetition, “−” no microbial growth observed.*

**TABLE 3:** Description of the strain isolates.

Isolated strain	Source material (plant photosynthetic mode and Cd treatment variant)	Colony morphology	MALDI-TOF identification (IF)	NCBI taxonomy identification number
S4	CAM 10 mM CdCl_2_	Milky white, round, sticky, medium	*Rhodococcus erythropolis* (1.81)	1833
S7	CAM 10 mM CdCl_2_	Light yellow, blurry edges, medium	*Paenibacillus glucanolyticus* (2.06)	159843
S10	CAM 10 mM CdCl_2_	Milky white, round, small	*Rhodococcus erythropolis* (1.87)	1833
W6	C_3_ 10 mM CdCl_2_	White, round, medium	*Providencia rettgeri* (2.48)	587
W7	C_3_ 10 mM CdCl_2_	Milky white, round, medium	*Providencia rettgeri* (2.31)	587

*IF, MALDI-TOF identification factor; scoring criteria: IF > 2.0: high confidence identification; 1.99 > IF > 1.70: low-confidence identification; IF < 1.69: no organism identification possible.*

### Microbial Population Dynamics at Variant Conditions

**FIGURE 7:**
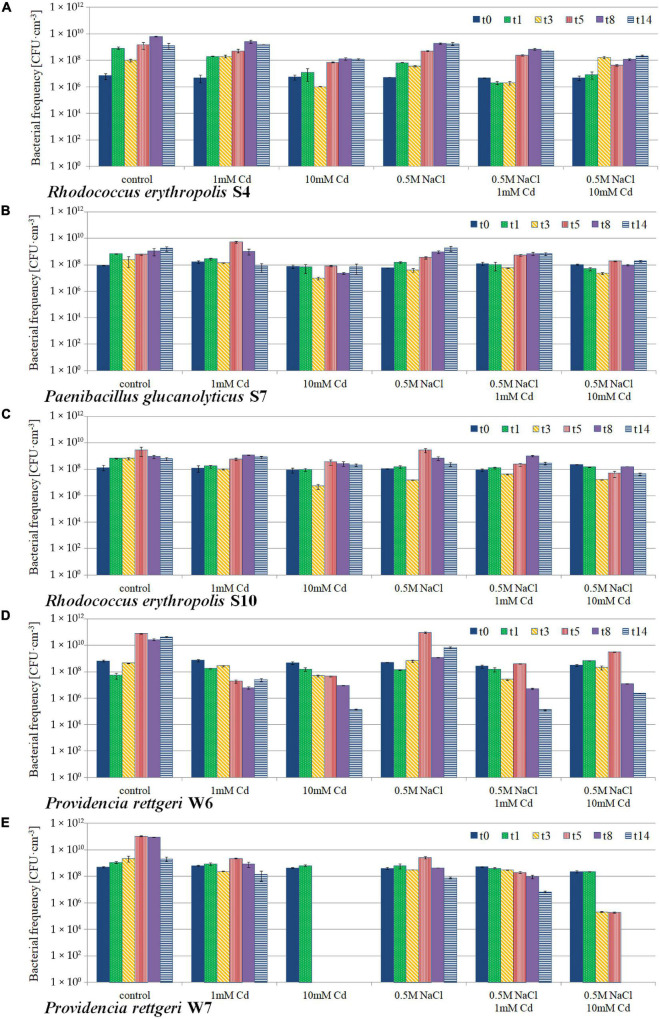
Population dynamics of Cd- and salt-tolerant bacterial isolates: **(A)** *Rhodococcus erythropolis* S4; **(B)** *Paenibacillus glucanolyticus* S7; **(C)** *Rhodococcus erythropolis* S10; **(D)** *Providencia rettgeri* W6; **(E)** *Providencia rettgeri* W7. The strains were isolated from the *M. crystallinum* rhizosphere upon 14-day cultivation in liquid media at variant conditions: control in the optimal growth medium; 1 mM Cd and 10 mM Cd, treatment with 1 mM and 10 mM CdCl_2_, respectively; 0.5 M NaCl, presence of 0.5 M NaCl in the medium; t0, start of the experiment; t1–t14, days of cultivation.

All five isolates were able to grow in the presence of CdCl_2_. The culture population dynamics of each particular strain isolate upon 14-day incubation at variant conditions is shown in Figure 7. *Rhodococcus erythropolis* S4 and S10 together with *Paenibacillus glucanolyticus* S7 were found to be resistant to 10 mM Cd both at the presence and absence of 0.5 M NaCl. For strains obtained from the rhizosphere of C_3_ plants, that is *Providencia rettgeri* isolates W6 and W7, a toxic effect of 10 mM CdCl_2_ was observed and these isolates revealed significant population decrease both in the presence and absence of NaCl. For W7, 10 mM cadmium toxicity was observed already after 24 h of the incubation and resulted either in the strong inhibition of growth in the presence of saline, or total survivability decrease in the absence of NaCl. Still, the strain could grow in the presence of NaCl and absence of Cd, but for that reason it has not been considered as a Cd-resistant one.

## Discussion

Based on the described extraordinary properties of the common ice plant and considering our recent data on its extreme resistance to cadmium and chromium (Nosek et al., 2019; Śliwa-Cebula et al., 2020), we have postulated *M. crystallinum* as a particularly favorable candidate for environmental biotechnology applications. For the case of heavy metal-contaminated saline areas, halophytes including the common ice plant can serve as potentially efficient phytoremediators (Wang et al., 2014; Lutts and Lefèvre, 2015), since plants tend to respond to both heavy metals and salinity by triggering similar physiological and biochemical mechanisms (Zhu, 2001).

The general rationale of the present work was to bring scientific proof supporting the idea of the involvement of rhizosphere microbiota in the overall response of the plant system to abiotic stress. In the experimental part we have tested *M. crystallinum* grown in normal and saline soils doped with cadmium under controlled laboratory conditions and then focused on the examination of microorganisms inhabiting the root zone. Depending on the salinity conditions (low or high salt supplementation), the plants revealed different photosynthetic modes, i.e., C_3_ or CAM, respectively.

The observed differences in growth and biomass formation between the C_3_ and CAM phases, manifested especially by reduced plant size and fresh mass under CAM, confirm recent observations of Guan et al. (2020), who noticed normal development of seedlings of *M. crystallinum* up to 6 days of NaCl treatment and then subsequent growth slowdown when compared to plants not treated with saline. On the other hand, no statistical differences were observed for each photosynthetic mode when plants were treated with different Cd concentrations supplemented to the soil. This observation agrees with our previous studies (Nosek et al., 2019; Śliwa-Cebula et al., 2020) in which we noticed that cadmium presence in the soil substrate did not affect biomass formation and significant plant morphological changes. Contrasting data were obtained by Ghnaya et al. (2005) who reported growth inhibition and substantial chlorosis upon plant cultivation on relatively smaller Cd doses (50 μM); however, the latter study was carried out in liquid nutrient solutions. It is to note that despite the experimental conditions, all the authors documented higher accumulation of cadmium in plant roots than in shoots.

The rhizosphere of the tested plants was highly colonized with microorganisms in all the experimental variants and showed considerable diversity based on colony morphotype evaluation even at the highest level of Cd administered (818 mg⋅kg^–1^ of soil d.w.). Recently, Kataoka et al. (2021) proved that the frequency of microorganisms was independent of the soil salinity. As regards isolation of pure strains, from the total of 21 rhizospheric isolates, nine proved to be fully cultivable under laboratory conditions and then five passed the Cd and saline toxicity test. Further testing revealed that bacteria inhabiting the root zones of CAM-mode plants (two identified as *Rhodococcus erythropolis* and one *Paenibacillus glucanolyticus*) exhibited the strongest tolerance to high cadmium concentrations, whereas two *Providencia rettgeri* strains obtained from the C_3_ phase (lack of NaCl administered to the soil) showed significantly weaker response.

*Paenibacillus glucanolyticus* S7, isolated from the rootzone of the CAM-performing common ice plant treated with 10 mM CdCl_2_, is a gram positive, rod shaped, facultative aerobic, motile bacillus with the potential of producing terminal spores (Alexander and Priest, 1989; Mathews et al., 2016). The species is relatively often obtained from root zones and from contaminated soils. It is known to synthesize enzymes participating in decomposition of celluloses, hemicelluloses, glucans, and lignin (Alexander and Priest, 1989; Mathews et al., 2016). Several researchers proved that *Paenibacillus* sp. strains could promote growth and development of plants, i.e., ornamental ones: gerbera and chrysanthemum (Zawadzka et al., 2013), rice (Banerjee et al., 2020), black pepper (Sangeeth et al., 2012), and crop (Grady et al., 2016). These bacteria were also shown to reveal antimicrobial and insecticide activities, to be capable of atmospheric nitrogen assimilation, solubilization of potash, production of IAA and other phytohormones, and release of siderophores enabling iron acquisition (Zawadzka et al., 2013; Grady et al., 2016).

Two *Rhodococcus erythropolis* strains (S4 and S10) were isolated from the rhizosphere of *M. crystallinum* treated with 10 mM Cd and saline solutions. It is a gram-positive, aerobic, non-sporulating, non-motile, and non-pathogenic Actinobacterium occurring in different environments including soils and plant tissues (Kim et al., 2018; Savory et al., 2020). There is evidence of positive impact of *Rh. erythropolis* on plant growth and biomass yield, mainly through solubilization of phosphate and zinc, and involvement in the mechanism of induction of systemic resistance (ISR) (Trivedi et al., 2007; Abraham and Silambarasan, 2018; Savory et al., 2020). The species is also known to possess unique and complex enzymatic arrays which enable adaptation and survival under harsh or extreme conditions (de Carvalho and da Fonseca, 2005). Moreover, it exhibits strong degrading activities toward different xenobiotics as well as resistance to heavy metals (Cejkova et al., 2005; Trivedi et al., 2007; de Carvalho, 2012; Abraham and Silambarasan, 2018). Interestingly, the growth of this microorganism in the presence of high concentrations (up to 5.5%) of NaCl was also reported (de Carvalho, 2012).

*Providencia rettgeri* strains W6 and W7 were identified in the root zone of the Cd-treated common ice plant, but only in the absence of salt supplementation. This is an unexpected result since this species is usually considered as tolerant to NaCl up to the concentration of 80 g⋅dm^–3^ (Benmalek et al., 2014). The species is a gram-negative, motile, aerobic short bacterium revealing nitrification properties (Abo-Amer et al., 2013; Ye et al., 2016). It is commonly present in the environment, both in waters and soils (Jackson et al., 1995). *Providencia* sp. strains were tested by Kartik et al. (2016) and their tolerance to elevated concentrations of Cd together with PGP effect were proven.

Abbas et al. (2018) stated that microbial populations are typically present in the environments contaminated with heavy metals and showed that they can tolerate toxic levels of these elements. To date, many authors isolated microorganisms from soils as well as other environments contaminated with cadmium. From among numerous strain isolates, the following ones are worth mentioning: *Comamonas testosteronii, Flavobacterium* sp., *Methylobacterium fujisawaense, Alcaligenes piechaudii* and *Alcaligenes xylosooxidans* (Kanazawa and Mori, 1996), *Klebsiella planticola* (Sharma et al., 2000), *Cupriavidus taiwanensis* and *Pseudomonas aeruginosa* (Siripornaldulsil and Siripornaldulsil, 2013), *Stenotrophomonas acidaminiphila* (Lin et al., 2016), *Providencia* spp. (Kartik et al., 2016), *Bacillus cereus, Alcaligenes faecalis* (El-Meihy et al., 2019), *Rhizobium pusense* (Li et al., 2019), *Enterobacter kobei*, and *Bacillus cereus* (Abdollahi et al., 2020), *Stenotrophomonas rhizophila, Variovorax boroniumulans*, and *Sporosarcina pasterurii* (Jalilvand et al., 2020). In the context of the above it is emphasized that the bacterial species isolated in the present study have never before been obtained from saline-rich and/or Cd-containing root zones of *Mesembryanthemum crystallinum* and to the best of our knowledge this is the first contribution to prove the occurrence of rhizospheric salt- and Cd tolerant strains of *Rhodococcus erythropolis*, *Paenibacillus glucanolyticus*, and *Providencia rettgeri*. Here, however, a strain identified as the genus *Paenibacillus* should be noted, which was isolated from *M. crystallinum* and described as a salt-tolerant endophytic bacterium (Zhang et al., 2018).

Within the common ice plant rhizosphere, favorable conditions for microbiota proliferation can be expected, which might possibly contribute to plant tolerance toward various stressors. For the case of toxic contaminants such as heavy metals including cadmium, the enhanced microbial activity is an important factor facilitating circulations of elements in soil, affecting their bioavailabilities and uptake by plants, and reducing their toxicities (Huang, 2004; Ali et al., 2013; Rizwan et al., 2017). Plant biostimulatory effect for the soil microbiota should be the most pronounced under the CAM phase. This is because the physiological and biochemical status of *M. crystallinum*, as described in detail elsewhere (Adams et al., 1998; Libik-Konieczny et al., 2019; Guan et al., 2020), may lead to the release of excess photosynthetic-borne oxygen through the root system directly to the soil. Then, enhanced production of two- and tricarboxylic acids such as malate and citrate, tend to be pumped out through the roots as soil-enriching exudates. As a result, growth of aerobic bacteria in the bulk soil of the rhizosphere is strongly promoted. Considering the above mechanisms together with the fact that the complex CAM metabolism is triggered by salinity treatment it can be assumed that the most potent Cd- and salt-tolerant strains should be obtainable from the root zone of CAM-performing plants. Indeed, our results support this idea as the strains *Rh. erythropolis* S4 and S10 and *P. glucanolyticus* S7 were found to be the most robust.

Based on the data collected so far, at the current stage it is difficult to elucidate a detailed mechanism of the interaction of the stress-evolved microbiota with the common ice plant. In this respect the key research problems are yet to be addressed and solved. They are related to the role of bacterial isolates as PGP agents and as microorganisms contributing to the *M. crystallinum* stress tolerance for heavy metals or salinization.

Summing up, we believe that *M. crystallinum* may owe its extreme cadmium resistance capabilities not only to its own biochemical properties and physiological reactions to stressors but also to the interactions with microorganisms inhabiting the root zone. Deeper understanding of a possible synergy of interactions on the level of plant rhizosphere—soil bacteria, while coping with harsh conditions of high salinity and severe Cd contamination, may be very important in terms of designing models for efficient phytoremediation with the common ice plant. Thus, further research has been planned to investigate the influence of the isolated bacteria on plant stress tolerance as well as to evaluate bacterial PGP properties. On the other hand, the isolates obtained from the rhizosphere of the CAM-performing plants are very promising microorganisms for potential use in environmental projects. They reveal unique characteristics enabling them to tolerate extreme Cd concentrations together with high salinity. Future studies will allow for applicability validation of these bacteria for bioremediation of saline sites affected by cadmium pollution.

## Data Availability Statement

The original contributions presented in the study are included in the article, further inquiries can be directed to the corresponding author.

## Author Contributions

ZM and PK designed the experiment. MŚ-C, PS, and PK performed the experiment, carried out the analyses, prepared figures and tables, performed statistical evaluation of the data, and wrote the original version of the manuscript. ZM was the project leader, acquired funding, and carried out the final review and editing. All the authors contributed to the article and approved the final version.

## Conflict of Interest

The authors declare that the research was conducted in the absence of any commercial or financial relationships that could be construed as a potential conflict of interest.

## Publisher’s Note

All claims expressed in this article are solely those of the authors and do not necessarily represent those of their affiliated organizations, or those of the publisher, the editors and the reviewers. Any product that may be evaluated in this article, or claim that may be made by its manufacturer, is not guaranteed or endorsed by the publisher.

## Footnotes

1
https://www.ncbi.nlm.nih.gov/Taxonomy/Browser/wwwtax.cgi
